# Appendicular Lean Mass Loss Does Not Impact Physical Performance Change During Caloric Restriction in Older Adults

**DOI:** 10.1093/geroni/igab046.301

**Published:** 2021-12-17

**Authors:** Daniel Beavers, Ryan Miller, Kristen Beavers, Barbara Nicklas

**Affiliations:** 1 Wake Forest School of Medicine, Winston-Salem, North Carolina, United States; 2 Wake Forest School of Medicine, Winston Salem, North Carolina, United States; 3 Wake Forest University, Winston Salem, North Carolina, United States

## Abstract

Data from 11 six-month randomized controlled trials were pooled, with 902 participants randomized to caloric restriction (CR; n=762) or Non-CR (n=140) to determine if CR-induced appendicular lean mass (ALM) loss was associated with change in physical performance among older adults. After adjusting for age, sex, race, body mass index, exercise assignment and baseline value of the outcome, CR had significant ALM loss [-0.77 kg (95% CI: -0.89, -0.65)], while Non-CR had ALM gain [+0.28 kg (0.08, 0.49)]; p<0.001. Both groups experienced similar improvements in the Short Physical Performance Battery (SPPB) score [CR: +0.45 (0.35, 0.55) vs Non-CR: +0.50 (0.30, 0.69); p=0.63] and sit-to-stand time [CR: -1.42 s (-1.81, -1.03) vs Non-CR: -1.85 s (-2.49, -1.21); p=0.19]. Change in SPPB score and sit-to-stand time was not associated with change in ALM (both p>0.15). In spite of significant ALM loss, CR resulted in overall improvements in physical performance in older adults.

